# Prior response to anti-VEGF agents predicts the efficacy of trifluridine/tipiracil plus bevacizumab in patients with metastatic colorectal cancer

**DOI:** 10.1007/s10147-026-03035-w

**Published:** 2026-04-30

**Authors:** Masahiro Hirakawa, Tamotsu Sagawa, Yutaka Okagawa, Norito Suzuki, Koshi Fujikawa, Kohichi Takada

**Affiliations:** 1 Division of Medical Oncology, Department of Internal Medicine, Sapporo Medical University School of Medicine, South 1, West 16, Chuo-ku, Sapporo, Hokkaido 060-8543 Japan; 2 Department of Gastroenterology, National Hospital Organization Hokkaido Cancer Center, Sapporo, Japan

**Keywords:** Trifluridine/tipiracil, Bevacizumab, Metastatic colorectal cancer, Predictive factor

## Abstract

**Background:**

Trifluridine/Tipiracil (FTD/TPI) combined with bevacizumab (Bev) is a standard treatment for patients with unresectable metastatic colorectal cancer (mCRC) in later-line therapy. Despite the availability of other drugs like regorafenib and fruquintinib, there is no consensus on the optimal treatment choice or sequence for later-line therapies due to a lack of clearly defined prognostic indicators. This multicenter real-world analysis aimed to identify predictive factors related to the effectiveness of FTD/TPI + Bev.

**Patients and methods:**

We retrospectively analyzed the medical records from our institution and collaborating centers of 71 mCRC patients given FTD/TPI + Bev as third-line or later treatment. Survival data were calculated using the Kaplan–Meier method, and group comparisons were made using the log-rank test. Multivariate analyses of progression-free survival (PFS) and overall survival (OS) were performed using a Cox proportional-hazards model.

**Results:**

A performance status (PS) of 0–1, absence of liver metastases, and fewer metastatic sites were significantly associated with improved PFS. For OS, significant differences were observed according to PS, presence of liver metastases, number of metastatic sites, and *RAS/BRAF* mutational status. Among patients who received anti-VEGF agents in second-line therapy, those with a second-line PFS of more than 9 months had significantly better median PFS and OS with FTD/TPI + Bev compared to those with a median PFS of 9 months or less. In multivariate analysis, PS and duration of second-line PFS were the only significant predictors of PFS and OS.

**Conclusion:**

The response to anti-VEGF agents in second-line therapy may predict the efficacy of FTD/TPI + Bev in subsequent treatments.

**Supplementary Information:**

The online version contains supplementary material available at 10.1007/s10147-026-03035-w.

## Introduction

Colorectal cancer (CRC) is the third most common cancer and its incidence is increasing worldwide. In 2022, around 1.9 million patients were newly diagnosed, and 903,859 patients died from CRC [1]. Although progress in targeted molecular therapies and combination approaches in the 1 st and 2nd line chemotherapy have significantly improved the prognosis for patients with unresectable metastatic colorectal cancer (mCRC) [2–4], effective utilization of agents in later lines of treatment is also crucial for further improving the prognosis. Trifluridine/Tipiracil (FTD/TPI) is an established treatment for mCRC in later-line therapy [5]. The SUNLIGHT trial demonstrated the benefits of adding bevacizumab (Bev) to FTD/TPI compared to FTD/TPI alone [6], leading to the widespread global adoption of FTD/TPI + Bev as a standard treatment in later-line therapy. Although other drugs, such as regorafenib and fruquintinib, are also indicated for mCRC [7, 8], a lack of clearly defined prognostic indicators means that there is currently no consensus on the optimal treatment choice or sequence for third- or later-line therapies. Therefore, we designed this multicenter real-world analysis to identify prognostic factors associated with the effects of FTD/TPI + Bev.

### Patients and methods

Clinical data from 71 patients with mCRC who received FTD/TPI + Bev as third- or later-line therapy between October 2018 and March 2025 were retrospectively collected from medical records at our institution and collaborating centers. Patients aged > 18 years with histologically confirmed mCRC were eligible.

FTD/TPI was administered orally at a dose of 70 mg/m^2^/day on days 1–5 and 8–12 every 28 days. Bev was administered intravenously at a dose of 5 mg/kg on day 1 and 15. Dose reductions were at the physician's discretion.　The study was approved by the institutional review board of Sapporo Medical University Hospital (approval number: 312–21) and was conducted in accordance with the Declaration of Helsinki (fourth edition). The requirement for written informed consent was waived because this was a retrospective analysis of routine data.

### Statistical analysis

Statistical analysis was conducted using GraphPad Prism 10.0 software or EZR (Jichi Medical University, Tochigi, Japan), which is a graphical user interface for R (The R Foundation for Statistical Computing, Vienna, Austria). Survival estimates were calculated using the Kaplan–Meier method for time-to-event analysis, and group comparisons were performed using the log-rank test. Multivariate analysis of progression-free survival (PFS) and overall survival (OS) were performed with the use of a Cox proportional-hazards model. A two-sided p-value < 0.05 was considered statistically significant.

## Results

### Patient characteristics

Seventy-one patients were included in our analysis (Table 1). The median age of the patients was 66 years, with 33 males and 38 females. The primary tumor was located on the right side in 25.3% of cases. Thirty-eight percent and 60.6% of the included patients had *RAS* wild-type and *RAS*/*BRAF*-mutant CRC, respectively. Thirty-two cases had metastasis in just one organ, whereas thirty-nine cases exhibited metastasis in two or more organs. A total of 57 patients (80.3%) had received anti-VEGF agents as part of their second-line therapy. Among patients receiving anti-VEGF therapy in the second-line setting, the agents administered were bevacizumab (28.2%), ramucirumab (49.3%), and aflibercept (2.8%). The distribution of treatment lines for FTD/TPI + Bev was as follows: 61 patients (85.9%) received the regimen as third-line therapy, 10 patients (14.1%) as fourth-line or later therapy.

**Table 1:** Patient characteristics

N=71	
Age, median (range)	66 (36–84)
Patient sex, N (%)	
Male	33 (46.5)
Female	38 (53.5)
ECOG performance status, N (%)	
0	40 (56.3)
1	20 (28.2)
2	10 (14.1)
≥3	1 (1.4)
Location of primary tumor, N (%)	
Right side	18 (25.4)
Left side	53 (74.6)
*RAS, BRAF* status, N (%)	
* RAS, BRAF *Wild type	27 (38.0)
* RAS* Mutated	43 (60.6)
* BRAF* V600E Mutated	1 (1.4)
Pathological diagnosis, N (%)	
Intestinal type	67 (94.4)
Diffuse type	4 (5.6)
Number of metastatic sites, N (%)	
1	32 (45.1)
≥2	39 (54.9)
Liver only disease, N (%)	12 (16.9)
Presence of liver metastasis, N (%)	
Yes	40 (56.3)
No	31 (43.7)
Administration of anti-VEGF agents in second-line therapy, N (%)	
Yes	57 (80.3)
Bevacizumab	20 (28.2)
Ramucirumab	35 (49.3)
Aflibercept	2 (2.8)
No	14 (19.7)
Second-line progression free survival, N (%)	
Patients who received anti-VEGF agents	
≤9 months	32 (45.1)
>9 months	25 (35.2)
Patients who did not receive anti-VEGF agents	
≤9 months	6 (8.5)
>9 months	8 (11.3)
Treatment lines for FTD/TPI+Bev	
3rd line	61 (85.9)
4th line or later	10 (14.1)

*ECOG* Eastern cooperative oncology group

### Favorable prognostic factors

**Fig. 1:**
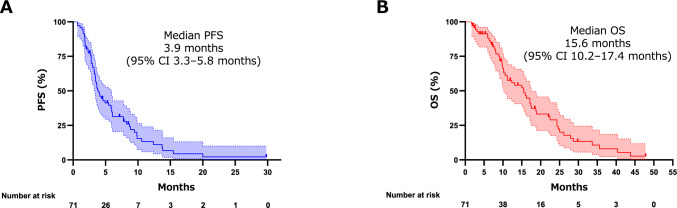
Kaplan–Meier plots depicting PFS (**A**) and OS (**B**) curves of all patients. PFS, progression-free survival; OS, overall survival; CI, confidence interval

**Fig. 2:**
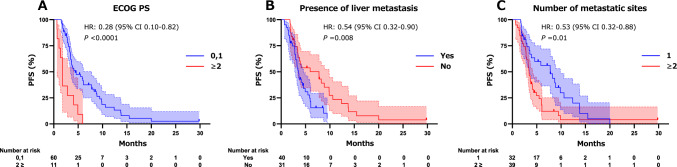
PFS according to each parameter. **A** ECOG PS (0,1 versus ≥ 2). **B** Presence of liver metastasis (Yes versus No). **C** Number of metastatic sites (1 versus ≥ 2)

**Fig. 3:**
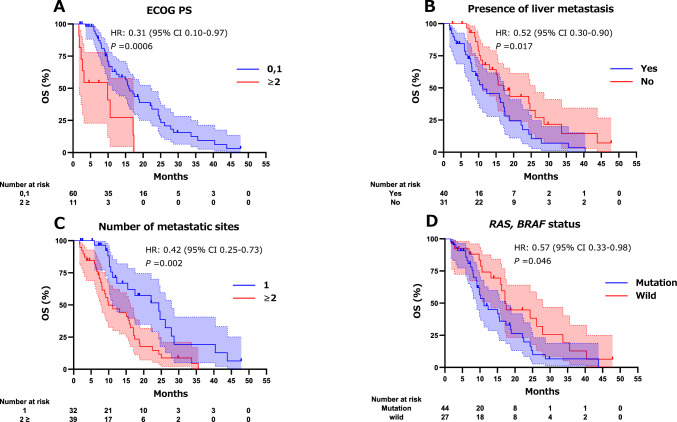
OS according to each parameter. **A** ECOG PS (0,1 versus ≥ 2). **B** Presence of liver metastasis (Yes versus No). **C** Number of metastatic sites (1 versus ≥ 2). **D** RAS, BRAF status (Mutation versus wild-type)

Overall response rate was 4.2%, and the disease control rate was 53.5% (Table 2). The median PFS was 3.9 months (95% CI 3.3–5.8 months) and the median OS was 15.6 months (95% CI 10.2–17.4 months) at a median follow-up time of 10.7 months (Fig. 1A, B). A performance status (PS) of 0 or 1, the absence of liver metastases, and a lower number of metastatic sites were significantly associated with improved PFS [PS 0, 1: 4.4 months (95% CI 3.4–7.6 months) vs. PS ≥ 2: 1.8 months (95% CI 0.7–4.1 months), HR 0.28 (95% CI 0.10–0.82; *P* < 0.0001), absence of liver metastases: 7.8 months (95% CI 3.4–9.8 months) vs. presence of liver metastases: 3.6 months (95% CI 2.8–4.6 months), HR 0.54 (95% CI 0.32–0.90; *P* = 0.008), one metastatic site: 7.8 months (95% CI 3.6–9.8 months) vs. number of metastatic sites ≥ 2: 3.4 months (95% CI 2.8–4.1 months), HR 0.53 (95% CI 0.32–0.88; *P* = 0.01)] (Fig. 2A, B, C). On the other hand, there were no significant differences in PFS based on age, *RAS*/*BRAF* mutational status, the primary tumor site, or the use or non-use of anti-VEGF agents in second-line therapy (Supplementary Figure S1A, B, C, D). With respect to OS, we observed statistically significant differences according to PS, the presence of liver metastases, the number of metastatic sites, and *RAS*/*BRAF* mutational status [PS 0, 1: 16.2 months (95% CI 11.2–22.5 months) vs. PS ≥ 2: 9.9 months (95% CI 1.8–17.1 months), HR 0.31 (95% CI 0.10–0.97; *P* = 0.0006), absence of liver metastases: 17.1 months (95% CI 11.1–26.1 months) vs. presence of liver metastases: 11.2 months (95% CI 8.0–17.4 months), HR 0.52 (95% CI 0.30–0.90; *P* = 0.017), one metastatic site: 24.3 months (95% CI 11.1–27.9 months) vs. number of metastatic sites ≥ 2: 9.9 months (95% CI 7.6–16.2 months), HR 0.42 (95% CI 0.25–0.73; *P* = 0.002), *RAS* wild type group: 17.1 months (95% CI 13.1–27.9 months) vs. *RAS*/*BRAF* mutation group: 11.2 months (95% CI 9.2–17.4 months), HR 0.57 (95% CI 0.33–0.98; *P* = 0.046)] (Fig. 3A, B, C, D). Conversely, no significant differences in OS were observed in relation to age under 65 years, the site of the primary tumor, or the administration of anti-VEGF agents in second-line therapy (Supplementary Figure S2A, B, C). In exploratory subgroup analyses stratified by treatment line, there were no significant differences in PFS or OS between patients receiving FTD/TPI + Bev as third-line therapy and those receiving it as later-line therapy [PFS: 3.7 months (95% CI 3.2–5.8 months) vs. 5.5 months (95% CI, 2.1–8.2 months), *p* = 0.68; OS: 15.6 months (95% CI, 10.1–18.7 months) vs. 15.0 months (95% CI, 3.1–33.6 months), *p* = 0.97] (Supplementary Figure S1E, S2D).

**Table 2:** Tumor response by RECIST version1.1

Best overall response, N (%)	
CR	0 (0)
PR	3 (4.2)
SD	35 (49.3)
DCR	38 (53.5)
PD	27 (38.0)
NA	6 (8.5)

*RECIST* Response evaluation criteria in solid tumors, *CR* Complete response, *PR* partial response, *SD* Stable disease, *DCR* (CR+PR+SD), Disease control rate, *PD* Progressive disease, *NA* Not available

### Impact of the response to prior anti-VEGF therapy

**Fig. 4:**
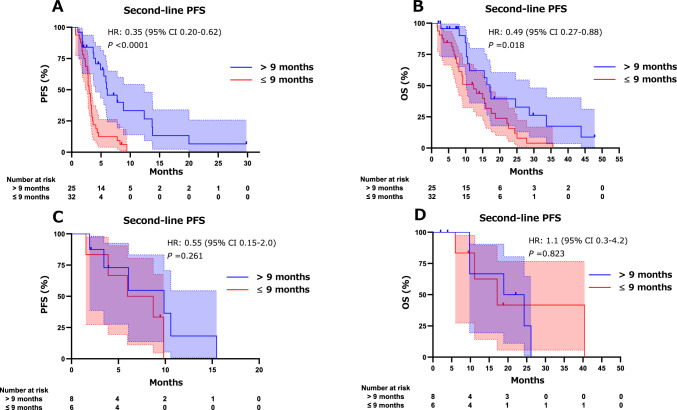
PFS and OS according to second-line PFS. PFS (**A**) and OS (**B**) in patients who received anti-VEGF agents in second-line (second-line PFS > 9 months versus ≤ 9 months). PFS (**C**) and OS (**D**) in patients who did not receive anti-VEGF agents in second-line (second-line PFS > 9 months versus ≤ 9 months)

To investigate whether the response to anti-VEGF agents in second-line therapy could predict the efficacy of FTD/TPI + Bev in later-line therapy, we further analyzed patients who received anti-VEGF agents in second-line therapy. Among these patients, those who had a second-line PFS of more than 9 months showed a significantly better median PFS with FTD/TPI + Bev compared to those with a median PFS of 9 months or less [second-line PFS > 9 months: 6.1 months (95% CI 4.1–12.4 months) vs. second-line PFS ≤ 9 months: 3.0 months (95% CI 2.3–3.4 months), HR 0.35 (95% CI 0.20–0.62; *P* < 0.0001)] (Fig. 4A). Similarly, patients with a second-line PFS of more than 9 months demonstrated a significantly improved OS when treated with FTD/TPI + Bev, in contrast to those with a median PFS of 9 months or less [second-line PFS > 9 months: 17.1 months (95% CI 10.2–28.6 months) vs. second-line PFS ≤ 9 months: 12.3 months (95% CI 7.3–15.9 months), HR 0.49 (95% CI 0.27–0.88; *P* = 0.018] (Fig. 4B).

**Fig. 5:**
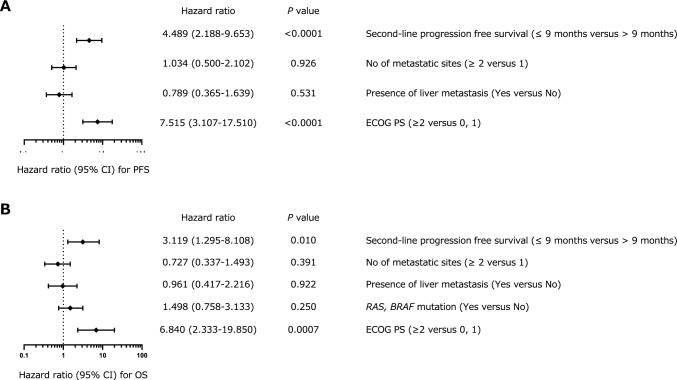
Multivariate analysis of PFS (**A**) and OS (**B**) in patients who received anti-VEGF agents in second-line

On the other hand, in patients who did not receive anti-VEGF agents in second-line therapy, the PFS of FTD/TPI + Bev showed no correlation with their second-line PFS [second-line PFS > 9 months: 9.9 months (95% CI 1.9–NA months) vs. second-line PFS ≤ 9 months: 7.3 months (95% CI 1.5–NA months), HR 0.55 (95% CI 0.15–2.0; *P* = 0.261] (Fig. 4C). There was also no correlation between the OS and second-line PFS in patients who did not receive anti-VEGF agents in second-line therapy [second-line PFS > 9 months: 21.6 months (95% CI 9.8–NA months) vs. second-line PFS ≤ 9 months: 17.1 months (95% CI 6.0–NA months), HR 1.1 (95% CI 0.3–4.2; *P* = 0,823] (Fig. 4D). Lastly, in multivariate analysis for patients who received anti-VEGF agents in second-line therapy, the only significant predictors of PFS and OS were PS and duration of second-line PFS (Fig. 5A, B).

## Discussion

The increasing treatment options for 3rd-line or later have improved the overall prognosis in mCRC [5–8]. However, there is no definitive consensus on the best choice and sequence of third-line or subsequent treatments, as there is a lack of sufficient evidence concerning the predictive factors for treatment efficacy associated with each therapy. Therefore, we conducted the current study to analyze real-world data from patients with mCRC receiving FTD/TPI + BEV, which is a promising treatment option for third- or later-line therapies, in order to elucidate the predictive factors of efficacy.

It is well known that mutations in *RAS*/*BRAF* are associated with poorer treatment outcomes when using epidermal growth factor receptor (EGFR) inhibitors such as cetuximab and panitumumab in mCRC patients [9–11]. Real world data from patients with mCRC receiving FTD/TPI monotherapy and furthermore, retrospective analyses of the RECOURSE trial suggested that *KRAS* codon G12 mutations may serve as potential biomarkers of resistance to FTD/TPI monotherapy in mCRC patients [12]. On the other hand, a meta-analysis of three RCTs—RECOURSE [5], TERRA [13], and J003 [14] which compared FTD/TPI to placebo indicates that FTD/TPI as a monotherapy for patients with *KRAS* G12 mutations provides an OS benefit, albeit this benefit may be less pronounced than that seen in patients without *KRAS* G12 mutations [15]. With respect to FTD/TPI + Bev, a post hoc analysis of the phase III SUNLIGHT trial revealed that its survival benefit was independent of *KRAS* mutational status [16]. Similarly, Doleschal et al. have shown that Bev may mitigate the potentially limited efficacy of FTD/TPI monotherapy in the *KRAS* G12-mutated population [17]. Besides the *KRAS* mutational status, several reports have been made regarding the predictive factors of treatment efficacy for FTD/TPI + Bev [18–20]. However, the decisive evidence for the optimal choice of third- or later-line treatments is still inadequate.

In our real-world retrospective analysis, a PS of 0 or 1, the absence of liver metastases, and a lower number of metastatic sites were associated with improved PFS. Regarding OS, a PS of 0 or 1, the absence of liver metastases, a lower number of metastatic sites and patients without *RAS/BRAF* mutations were associated with improved outcome. These findings largely reflect the impact of general prognostic factors such as functional status and tumor burden rather than treatment-specific effects. Importantly, the distribution of treatment lines (third-line versus later-line therapy) was heterogeneous within our cohort, which may have influenced survival outcomes. However, this heterogeneity reflects real-world clinical practice and underscores the need to identify pragmatic indicators that to guide treatment selection beyond line of therapy alone.

We also examined whether the prior use of anti-VEGF agents affected the efficacy of FTD/TPI + Bev. However, consistent with a previous report [21], we found no significant difference in the treatment efficacy of FTD/TPI + Bev regardless of prior use of anti-VEGF agents.

In the ML18147 trial, which investigated continued use of Bev plus standard second-line chemotherapy in patients with mCRC progressing after standard first-line Bev-based treatment, the OS tended to be shorter in the group with a PFS of less than 9 months during first-line therapy compared to those with a PFS of 9 months or more [22]. Therefore, we analyzed whether the response to anti-VEGF agents in second-line treatment could serve as a predictive factor for the efficacy of FTD/TPI + Bev in third-line or later therapy. As expected, PFS and OS were significantly better in patients with a PFS of more than 9 months during second-line therapy, and this statistical significance was maintained in the multivariate analysis. The choice of a 9-month PFS cutoff warrants careful consideration. This threshold was originally derived from the ML18147 trial in the context of first-line bevacizumab-based therapy, and PFS is generally shorter in later treatment lines. Therefore, patients achieving a second-line PFS of ≥ 9 months likely represent a subgroup with intrinsically favorable disease biology. Indeed, in our cohort, longer second-line PFS was correlated with other favorable characteristics, including better PS and lower metastatic burden. Consequently, our findings should not be interpreted as evidence that prolonged benefit from second-line anti-VEGF therapy directly enhances the subsequent efficacy of FTD/TPI plus bevacizumab, but rather that sensitivity to anti-VEGF-based therapy may serve as a surrogate marker of a favorable-risk population more likely to benefit from continued VEGF inhibition. This interpretation is further supported by the observation that, in patients who did not receive anti-VEGF agents during second-line therapy, no significant association was found between second-line PFS and outcomes with FTD/TPI + Bev. Given the relatively small sample size of this subgroup, limited statistical power cannot be excluded, and these results should be interpreted with caution. Larger datasets will be required to clarify whether second-line PFS retains predictive value in the absence of prior anti-VEGF exposure.

From a clinical perspective, these findings raise important considerations regarding treatment sequencing in later-line mCRC. If prolonged disease control with prior therapy primarily reflects favorable tumor biology and lower disease burden, similar benefits may also be achievable with other approved later-line agents, such as regorafenib or fruquintinib. Therefore, second-line PFS and baseline prognostic factors should be viewed as tools to guide individualized treatment selection rather than as determinants of a single optimal regimen. Comparative prospective studies will be necessary to establish whether specific patient subgroups derive preferential benefit from FTD/TPI + Bev compared with other later-line options.

Finally, it should be noted that anti-VEGF agents used in second-line therapy were not limited to bevacizumab in all cases. Different anti-VEGF agents, such as ramucirumab or aflibercept, exert distinct effects on angiogenic signaling pathways, which may influence subsequent responses to VEGF inhibition. Owing to the retrospective nature of this study and the limited sample size, we were unable to perform robust subgroup analyses according to the specific anti-VEGF agent used. This represents an additional limitation and an important area for future investigation.

In summary, our real-world data indicate that general prognostic factors and the duration of disease control achieved with prior anti-VEGF-based therapy are associated with outcomes of FTD/TPI + Bev in later-line mCRC. These findings may provide practical guidance for treatment sequencing and highlight the need for further studies to refine predictive markers for later-line therapeutic strategies.

## Conclusion

Good PS, the absence of liver metastases, and a lower number of metastatic sites were associated with better PFS and OS in patients with mCRC receiving FTD/TPI + Bev. Furthermore, the responsiveness to anti-VEGF agents in second-line therapy may predict the efficacy of FTD/TPI + Bev in third-line or later therapy.

## Supplementary Information

Below is the link to the electronic supplementary material.

Supplementary file1 (PPTX 1003 KB)

## Data Availability

The data are available from the corresponding author upon reasonable request.
