# Supplementary material for: Prior response to anti-VEGF agents predicts the efficacy of trifluridine/tipiracil plus bevacizumab in patients with metastatic colorectal cancer
Source: Int J Clin Oncol. 2026 Apr 30;31(7):1267–74. doi: 10.1007/s10147-026-03035-w (PMC13303470; doi:10.1007/s10147-026-03035-w)
Supplement: Supplementary file 1 — Supplementary file1 (PPTX 1003 KB) [file 10147_2026_3035_MOESM1_ESM.pptx]

## Slide 1
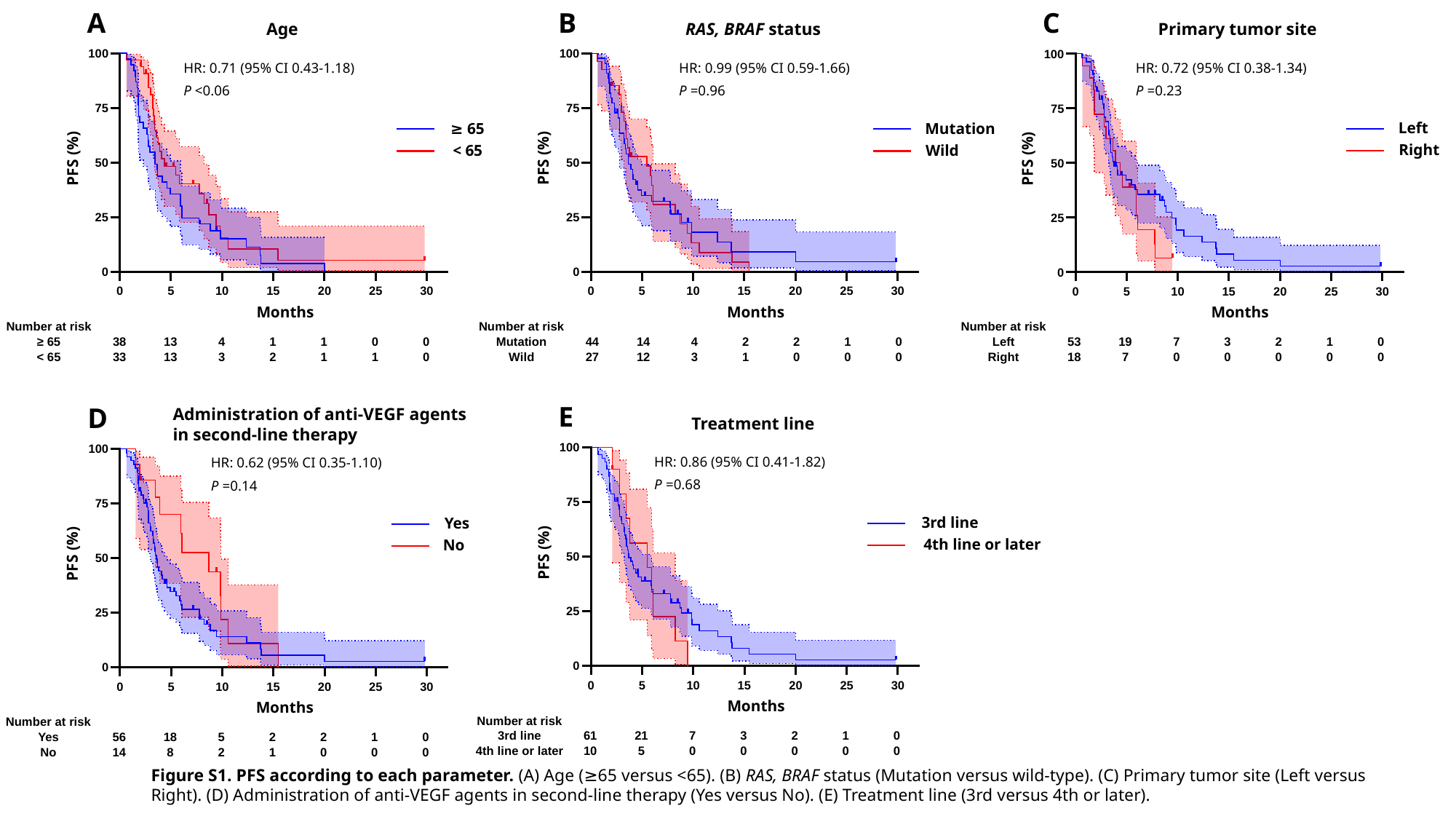

A
B
C
Age
RAS, BRAF status
Primary tumor site
| HR: 0.71 (95% CI 0.43-1.18) |
| --- |
| P <0.06 |
| HR: 0.99 (95% CI 0.59-1.66) |
| --- |
| P =0.96 |
| HR: 0.72 (95% CI 0.38-1.34) |
| --- |
| P =0.23 |
Left
≥ 65
Mutation
Right
< 65
Wild
PFS (%)
PFS (%)
PFS (%)
Months
Months
Months
| Number at risk | | | | | | | |
| --- | --- | --- | --- | --- | --- | --- | --- |
| Mutation | 44 | 14 | 4 | 2 | 2 | 1 | 0 |
| Wild | 27 | 12 | 3 | 1 | 0 | 0 | 0 |
| Number at risk | | | | | | | |
| --- | --- | --- | --- | --- | --- | --- | --- |
| ≥ 65 | 38 | 13 | 4 | 1 | 1 | 0 | 0 |
| < 65 | 33 | 13 | 3 | 2 | 1 | 1 | 0 |
| Number at risk | | | | | | | |
| --- | --- | --- | --- | --- | --- | --- | --- |
| Left | 53 | 19 | 7 | 3 | 2 | 1 | 0 |
| Right | 18 | 7 | 0 | 0 | 0 | 0 | 0 |
E
D
Administration of anti-VEGF agents
in second-line therapy
Treatment line
| HR: 0.86 (95% CI 0.41-1.82) |
| --- |
| P =0.68 |
| HR: 0.62 (95% CI 0.35-1.10) |
| --- |
| P =0.14 |
3rd line
Yes
4th line or later
No
PFS (%)
PFS (%)
Months
Months
| Number at risk | | | | | | | |
| --- | --- | --- | --- | --- | --- | --- | --- |
| 3rd line | 61 | 21 | 7 | 3 | 2 | 1 | 0 |
| 4th line or later | 10 | 5 | 0 | 0 | 0 | 0 | 0 |
| Number at risk | | | | | | | |
| --- | --- | --- | --- | --- | --- | --- | --- |
| Yes | 56 | 18 | 5 | 2 | 2 | 1 | 0 |
| No | 14 | 8 | 2 | 1 | 0 | 0 | 0 |
Figure S1. PFS according to each parameter. (A) Age (≥65 versus <65). (B) RAS, BRAF status (Mutation versus wild-type). (C) Primary tumor site (Left versus Right). (D) Administration of anti-VEGF agents in second-line therapy (Yes versus No). (E) Treatment line (3rd versus 4th or later).

## Slide 2
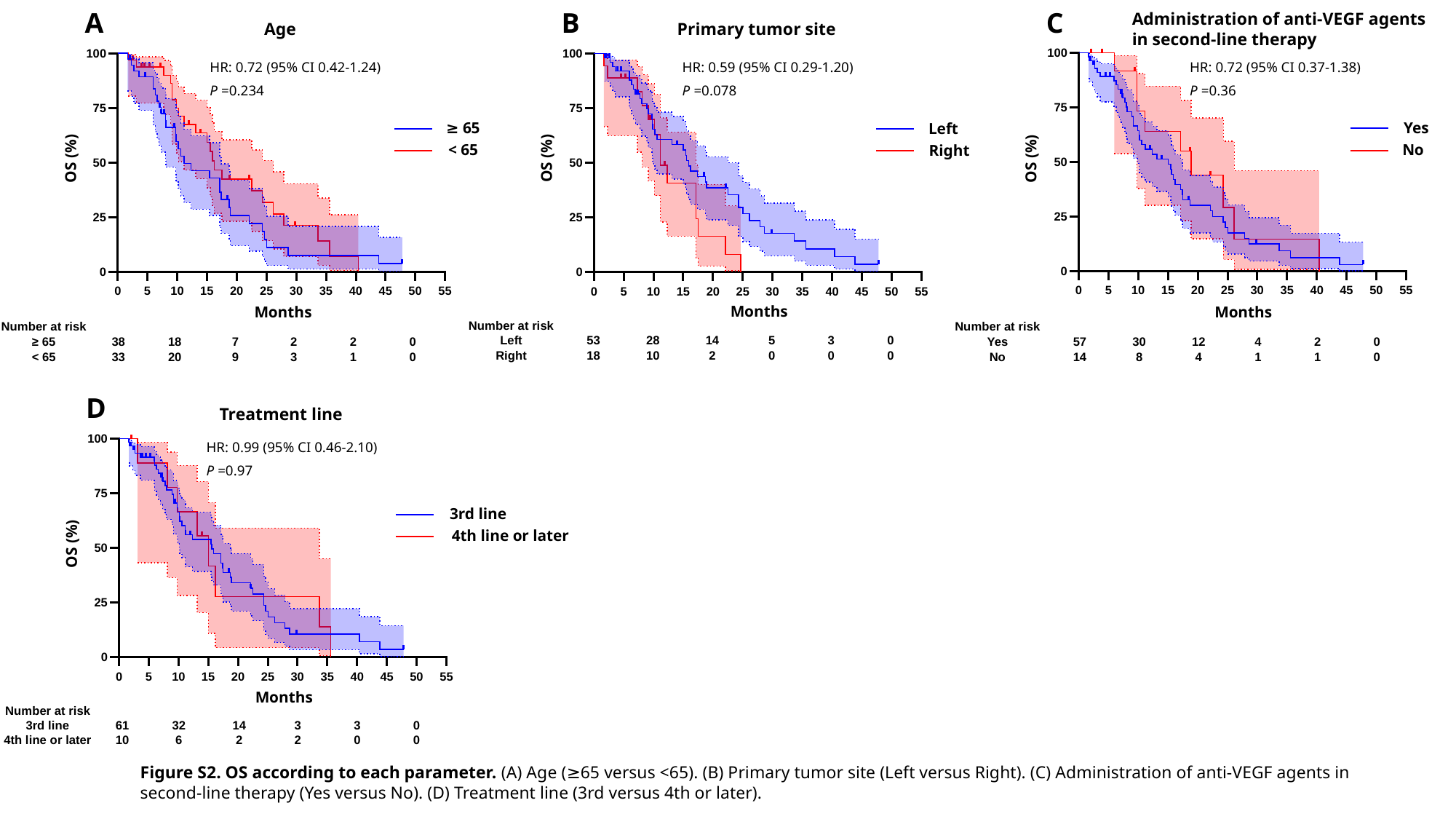

A
B
C
Administration of anti-VEGF agents
in second-line therapy
Age
Primary tumor site
| HR: 0.72 (95% CI 0.42-1.24) |
| --- |
| P =0.234 |
| HR: 0.59 (95% CI 0.29-1.20) |
| --- |
| P =0.078 |
| HR: 0.72 (95% CI 0.37-1.38) |
| --- |
| P =0.36 |
Yes
≥ 65
Left
No
< 65
Right
OS (%)
OS (%)
OS (%)
Months
Months
Months
| Number at risk | | | | | | |
| --- | --- | --- | --- | --- | --- | --- |
| Left | 53 | 28 | 14 | 5 | 3 | 0 |
| Right | 18 | 10 | 2 | 0 | 0 | 0 |
| Number at risk | | | | | | |
| --- | --- | --- | --- | --- | --- | --- |
| Yes | 57 | 30 | 12 | 4 | 2 | 0 |
| No | 14 | 8 | 4 | 1 | 1 | 0 |
| Number at risk | | | | | | |
| --- | --- | --- | --- | --- | --- | --- |
| ≥ 65 | 38 | 18 | 7 | 2 | 2 | 0 |
| < 65 | 33 | 20 | 9 | 3 | 1 | 0 |
D
Treatment line
| HR: 0.99 (95% CI 0.46-2.10) |
| --- |
| P =0.97 |
3rd line
4th line or later
OS (%)
Months
| Number at risk | | | | | | |
| --- | --- | --- | --- | --- | --- | --- |
| 3rd line | 61 | 32 | 14 | 3 | 3 | 0 |
| 4th line or later | 10 | 6 | 2 | 2 | 0 | 0 |
Figure S2. OS according to each parameter. (A) Age (≥65 versus <65). (B) Primary tumor site (Left versus Right). (C) Administration of anti-VEGF agents in second-line therapy (Yes versus No). (D) Treatment line (3rd versus 4th or later).
